# Maternal and infant morbidity following administration of repeat dexamethasone or betamethasone prior to preterm birth: A secondary analysis of the ASTEROID Trial

**DOI:** 10.1371/journal.pone.0263927

**Published:** 2022-02-22

**Authors:** Olivia J. Hofer, Jane E. Harding, Thach Tran, Caroline A. Crowther

**Affiliations:** 1 Faculty of Medical and Health Sciences, University of Auckland, Auckland, New Zealand; 2 Liggins Institute, University of Auckland, Auckland, New Zealand; 3 Osteoporosis and Bone Biology, Garvan Institute of Medical Research, Sydney, NSW, Australia; Centre Hospitalier Departementai Vendee, FRANCE

## Abstract

**Background:**

Clinical practice guidelines recommend administering antenatal corticosteroids (ACS), either betamethasone or dexamethasone, to women at risk of preterm birth at less than 35 weeks’ gestation. If women remain at risk of preterm birth seven or more days after an initial course of ACS, most guidelines recommend administration of a repeat dose(s). No randomised trials have assessed the efficacy of dexamethasone as a repeat steroid compared to betamethasone.

**Aim:**

We aimed to determine if there were differences between the use of dexamethasone or betamethasone as repeat ACS, for women who remain at risk of preterm birth after an initial course, on maternal, infant, and childhood health outcomes.

**Methods:**

We performed a secondary analysis of data from the ASTEROID randomised trial, where women at risk of preterm birth were allocated to either betamethasone or dexamethasone. Infant, childhood, and maternal outcomes were compared according to whether women received a repeat dose(s) of dexamethasone or betamethasone. The primary outcome was a composite outcome of death or any neurosensory disability at age two years (corrected for prematurity). The ASTEROID trial is registered with ANZCTR, ACTRN12608000631303.

**Results:**

168 women and their infants were included, with 86 women receiving dexamethasone and 82 women receiving betamethasone as a repeat dose. Women in the two ACS groups had similar baseline characteristics. We observed little to no difference in the incidence of death or any neurosensory disability at age two years (OR 0.89, 95% CI 0.39 to 2.06, p = 0.79) or in the incidence of other infant, childhood, and maternal adverse health outcomes between women who received dexamethasone and those who received betamethasone.

**Conclusion:**

Use of dexamethasone for a repeat dose(s) compared to betamethasone did not result in any differences in infant, childhood, and maternal health outcomes. These results can be used to support clinical practice guideline recommendations.

## Introduction

The administration of antenatal corticosteroids (ACS) to women prior to preterm birth before 35 weeks’ gestation improves infant health by accelerating the development of the lungs and other organ systems, thus reducing infant mortality and morbidity, including respiratory distress syndrome and intraventricular haemorrhage [1]. Clinical practice guidelines recommend using 24 mg of a corticosteroid, either dexamethasone or betamethasone, given in a divided dose over a 24 hour period [2–5]. For women who remain at risk of preterm birth seven or more days after an initial course of antenatal corticosteroids, most clinical practice guidelines recommend administration of a repeat dose(s) of ACS when preterm birth is planned or expected within the next seven days [2, 3, 5].

All the randomised trials published to date assessing the efficacy of repeat ACS have compared betamethasone with placebo or no treatment [6]. Even after an extensive search, a meta-analysis found no randomised trials that had used dexamethasone [6]. In both aggregate [6] and IPD metanalyses [7] (n = 4,857 women and 5,915 infants), when women at risk of preterm birth received a repeat dose(s) of betamethasone, a decreased risk of requiring respiratory support was found for their infants compared to infants of women who did not receive a repeat dose(s).

Given that most clinical practice guidelines [2, 3, 5] recommend using dexamethasone or betamethasone prior to preterm birth, it is important to determine the efficacy of dexamethasone when repeat ACSs are clinically indicated.

With no evidence to date from randomised trials regarding the efficacy of dexamethasone for repeat dose(s) compared to betamethasone, and calls for further research [6], we performed a secondary analysis of the ASTEROID Trial cohort [8]. We aimed to determine if there were differences in maternal, infant, and childhood health outcomes when dexamethasone was compared with betamethasone as repeat ACS in women who remained at risk of preterm birth after an initial course of ACS.

## Materials and methods

This study is a secondary analysis of data from the two-arm, parallel, double-blind ASTEROID Trial, in which women at risk of preterm birth at <34 weeks’ gestation from 14 maternity hospitals in Australia and New Zealand were allocated to either a course of dexamethasone (12 mg, two doses, 24 hours apart) or betamethasone (11.4 mg, two doses, 24 hours apart) between January 28th, 2009 and February 1st, 2013 [8]. A repeat single dose of the same ACS could be given at weekly intervals up to a maximum of three repeat doses to 32 weeks’ gestation if a woman was considered by her attending clinician to be at ongoing risk of preterm birth within the next seven days. Participants, clinical staff and those assessing the study outcomes remained masked to the treatment allocation. The Human Research Ethics Committee at the Women’s and Children’s Hospital, Adelaide, approved this research (REC2074/7/14). Informed written consent was obtained for each participant.

Women with a singleton pregnancy were eligible for inclusion in this secondary analysis if they had participated in the ASTEROID Trial, and they received a repeat dose(s) of the study drug (dexamethasone or betamethasone).

Antenatal, birth, postnatal, and infant health outcomes in infants up to the time of leaving the hospital after birth were retrieved from the medical records by the research staff. The childhood outcomes at two years corrected age were assessed by a paediatrician, psychometrist, and questionnaires completed by the child’s caregivers. These data were de-identified and used in this analysis.

Our study outcomes were those used in the ASTEROID Trial [8]. The primary outcome was a composite outcome of death or any neurosensory disability at age two years (corrected for prematurity). The composite outcome was defined as stillbirth, death of a live born infant either before or after hospital discharge, or any neurosensory disability (cerebral palsy, blindness, deafness, or developmental [cognitive, language or motor] delay, as determined in assessments by the paediatrician and psychometrist) [8].

The secondary outcomes for infants before hospital discharge were gestational age at birth, intraventricular haemorrhage (IVH), severe IVH (grade 3 or 4), periventricular leukomalacia (PVL), retinopathy of prematurity receiving treatment (ROP), patent ductus arteriosus receiving treatment (PDA), respiratory distress syndrome (RDS), severity of neonatal lung disease, chronic lung disease (defined as a need for oxygen supplementation at 36 weeks postmenstrual age or 28 days after birth, if born after 32 weeks of gestation) [8], use of mechanical ventilation, confirmed infection within the first 48 hours after birth, infection after the first 48 hours after birth, necrotising enterocolitis (NEC), admission to the neonatal intensive care unit (NICU), length of stay in NICU and, body size and z scores at birth (weight, length and head circumference) and at discharge from the hospital [8].

The secondary outcomes for children at two years (corrected for prematurity) were death or major neurosensory disability (defined as severe or moderate disability, including developmental delay with a standardised score more than 2 SD below the mean, cerebral palsy in a child who was not ambulant by age two years, blindness, or deafness), individual components, body size, general health (including use of health services since leaving the hospital after birth), childhood respiratory morbidity, blood pressure and Z scores, the proportion of results in hypertensive ranges, and child behaviour [8].

Maternal outcomes included perinatal infectious morbidity (defined as clinical chorioamnionitis that required intrapartum antibiotics and use of postpartum antibiotics, or both), induction of labour, mode of birth (vaginal or caesarean), postpartum haemorrhage, blood transfusion required and duration of postpartum hospital stay [8].

We first performed unadjusted analyses and then adjusted for the stratification factors of hospital site and gestational age at trial entry (<28 weeks or ≥28 weeks of gestation), i.e., the fixed effects. For the 2-year outcomes, we also adjusted for the language spoken at home, the mother’s education, and the sex of the child.

We analysed binary outcomes with logistic regression and treatment effects are expressed as odds ratios and 95% confidence intervals (CI). For rare outcomes, we performed a Fisher’s exact test. We used linear regression to determine the effect of the treatment group on continuous outcomes and these are expressed as differences in means. Treatment effects of count outcomes are expressed as ratios of means using log-Poisson regression or negative binomial regression when we found overdispersion. Proportional odds models were used to analyse ordinal outcomes and treatment effects are expressed as odds ratios of higher severity. Where the proportional odds assumption was not met, we used separate logistic regression for binary outcomes defined by different cut points, in which treatment effects are expressed as odds ratios. We did not adjust for multiple comparisons. We considered a two-sided p-value of less than 0.05 to indicate significance. We used JMP 15 (SAS Institute, Inc., Cary, NC) for our statistical analyses. The ASTEROID Trial is registered with ANZCTR, ACTRN12608000631303.

## Results

**Fig 1:**
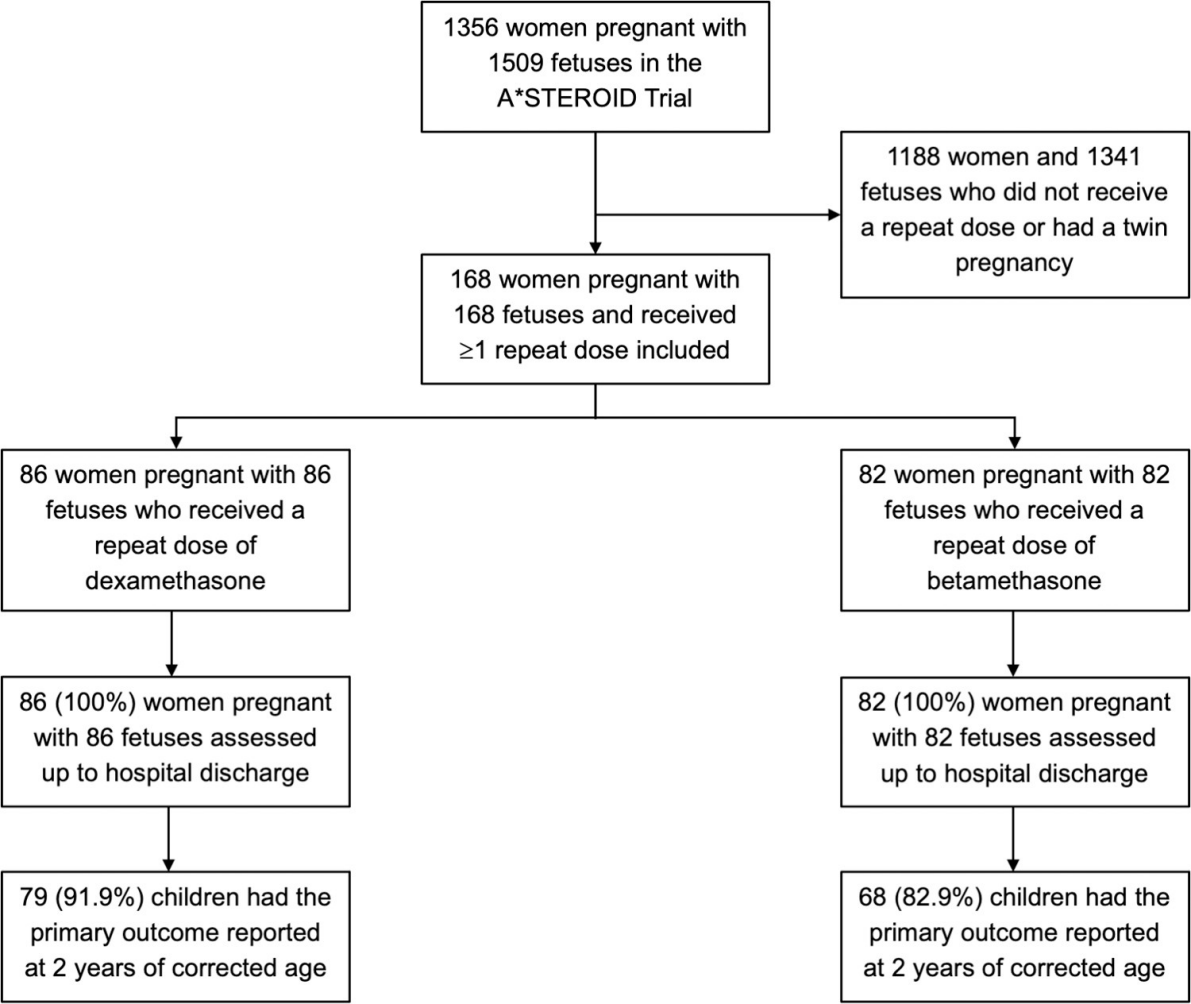
Women and infants included in study population.

A total of 168 women and their infants were eligible and therefore included in this secondary analysis; 86 in the dexamethasone and 82 in the betamethasone group (Fig 1). The demographic and pregnancy characteristics of women allocated dexamethasone were similar to those receiving betamethasone, although women who were allocated dexamethasone had on average a lower body mass index and fewer were obese (Table 1). A total of 96 women (57.1%) received their first dose of antenatal corticosteroids at less than 28 weeks’ gestation. The main reason for preterm birth in both treatment groups was preterm prelabour rupture of membranes, followed by antepartum haemorrhage. Over half the women received only one repeat dose of antenatal corticosteroids (99/168, 58.9%), 17.9% received two repeat doses and 23.2% received 3 repeat doses of corticosteroids.

**Table 1:** Maternal, infant and childhood characteristics of the cohort.

Characteristics	Total (N = 168)	Dexamethasone (N = 86)	Betamethasone (N = 82)	P-value
**Maternal Characteristics**				
Nulliparous	61 (36.3%)	31 (36.1%)	30 (36.6%)	0.94
Main ethnicity				0.62
European	135 (80.4%)	67 (77.9%)	68 (82.9%)	
Asian	19 (11.3)	11 (12.8)	8 (9.8%)	
Aboriginal or Torres Strait Islanders	2 (1.2%)	2 (2.3)	0 (0.0%)	
Polynesian	1 (0.6%)	0 (0.0%)	1 (1.2%)	
Māori	2 (1.2%)	1 (1.2%)	1 (1.2%)	
Other	9 (5.4%)	5 (5.8%)	4 (4.9%)	
Body mass index, kg/m2	27.3 (6.6)	25.9 (5.3)	28.1 (7.4)	0.03
Body mass index categories				0.23
Underweight (<18 kg/m2)	3 (2.3%)	1 (1.6%)	2 (3.0%)	
Normal (18–25 kg/m2)	54 (41.2%)	31 (48.4%)	23 (34.3%)	
Overweight (25–30 kg/m2)	33 (25.2%)	17 (26.6%)	16 (23.9%)	
Obese (> 30 kg/m2)	41 (31.3%)	15 (23.4%)	26 (38.8%)	
Gestational age at entry,:				0.72
<28 weeks’ gestations	96 (57.1%)	48 (55.8%)	48 (58.5%)	
≥28 weeks’ gestation	72 (42.9%)	38 (44.2%)	34 (41.5%)	
Maternal age	31.2 (6.4)	30.6 (6.3)	31.7 (6.5)	0.27
Previous preterm birth (<37 weeks’ gestation)	53 (49.5%)	24 (43.6%)	29 (55.8%)	0.21
Previous perinatal deaths (≥20 weeks’ gestation)	20 (11.9%)	9 (10.5%)	11 (13.4%)	0.56
Main reason for preterm birth				0.60
Antepartum haemorrhage	37 (22.0%)	22 (25.6%)	15 (18.3%)	
Preterm prelabour rupture of membranes	42 (25.0%)	22 (25.6%)	20 (24.4%)	
Preterm labour	18 (10.7%)	8 (9.3%)	10 (12.2%)	
Cervical incompetence	27 (16.1%)	12 (14.0%)	15 (18.3%)	
Pre-eclampsia	21 (12.5%)	10 (11.6%)	11 (13.4%)	
Fetal compromise	15 (8.9%)	6 (7.0%)	9 (11.0)	
Other	8 (4.8%)	6 (7.0%)	2 (2.4%)	
Number of repeat doses				0.20
1 repeat dose	99 (58.9%)	53 (61.6%)	46 (56.1%)	
2 repeat doses	30 (17.9%)	11 (12.8%)	19 (23.2%)	
3 repeat doses	39 (23.2%)	22 (25.6%)	17 (20.7%)	
**Infant Characteristics**				
Gestation at birth	33.2 (3.7)	33.2 (3.6)	33.2 (3.9)	1.00
Female sex	88 (52.4%)	46 (53.5%)	42 (51.2%)	0.77

Data are N (%) or mean (SD). P value from chi-squared test or student’s t-Test.

The incidence of the primary outcome of death or any neurosensory disability at age two years (corrected for prematurity) was similar in the dexamethasone group (24 of 79 infants, 30.4%) and the betamethasone group (20 of 68 infants, 32.4%), (adjusted odds ratio (aOR) 0.89, 95% CI 0.39 to 2.06, p = 0.79) (Table 2). We performed an exploratory analysis to assess the impact of maternal BMI on our primary outcomes due to the difference observed between the treatment groups. We observed a similar incidence of the primary outcome after adjusting for maternal BMI (aOR 0.82, 95% CI 0.31 to 2.18, p = 0.70).

**Table 2:** Type of antenatal corticosteroid and primary and secondary childhood outcomes at 2 years.

	Dexamethasone N, %	Betamethasone N, %	Unadjusted treatment effect (95% CI)	Unadjusted P-value	Adjusted treatment effect (95% CI)	Adjusted P-value
**Primary outcome**						
Death or any neurosensory disability	24/79 (30.4%)	20/68 (32.4%)	0.91 (0.45, 1.83)	0.80	0.89 (0.39, 2.06)	0.79
**Secondary outcomes**						
Death or major neurosensory disability	11/77 (14.3%)	10/68 (15.2%)	0.93 (0.37, 2.36)	0.88	1.06 (0.35, 3.18)	0.92
Any death	2/86 (2.3%)	3/82 (3.7%)	-	0.67 *	-	-
Stillbirth	0/86 (0.0%)	0/82 (0.0%)	-	-	-	-
Death of live born infant before hospital discharge†	2/86 (2.3%)	2/82 (2.4%)	-	1.00 *	-	-
Death after discharge	0/86 (0.0%)	1/82 (1.2%)	-	0.49 *	-	-
Any neurosensory disability	22/77 (28.6%)	19/65 (29.2%)	0.97 (0.47, 2.00)	0.93	0.91 (0.36, 2.16)	0.83
Blindness	0/80 (0.0%)	0/68 (0.0%)	-	-	-	-
Deafness	4/80 (5.0%)	3/68 (4.4%)	-	1.00 *	-	-
Cerebral palsy (definite or probable diagnosis by paediatric assessment)	2/80 (2.5%)	0/67 (0.0%)	-	0.50 *	-	-
Severity of cerebral palsy §						
Mild	0/80 (0.0%)	0/67 (0.0%)	-	-	-	-
Moderate	0/80 (0.0%)	0/67 (0.0%)	-	-	-	-
Severe	2/80 (2.5%)	0/67 (0.0%)	-	0.50*	-	-
Developmental delay						
Cognitive or language developmental delay	17/78 (21.8%)	17/66 (25.8%)	0.80 (0.37, 1.74)	0.58	0.70 (0.27, 1.80)	0.46
Severity of cognitive or language developmental delay ¶						
Mild	12/78 (15.4%)	11/66 (16.7%)	0.80 (0.37, 2.74)	0.58	0.70 (0.27, 1.80)	0.46
Moderate *	4/78 (5.1%)	3/66 (4.6%)	-	0.75	-	-
Severe	1/78 (1.3%)	3/66 (4.6%)	-	0.33*	-	-
Motor developmental delay||	7/77 (9.1%)	12/67 (17.9%)	0.46 (0.17, 1.24)	0.13	0.35 (0.10, 1.19)	0.09
Severity of motor developmental delays ¶						
Mild	6/77 (7.8%)	8/67 (11.9%)	0.46 (0.17, 2.34)	0.13	0.35 (0.10, 1.19)	0.09
Moderate	0/77 (0.0%)	2/67 (3.0%)	-	0.18*	-	-
Severe	1/77 (1.3%)	2/67 (3.0%)	-	0.60*	-	-
BSID-III						
Cognitive score	99.5 (14.8)	98.1 (18.3)	1.44 (-3.96, 6.84)	0.60	1.79 (-3.69, 7.27)	0.52
Language score	97.2 (17.9)	97.8 (20.1)	-0.52 (-6.80, 5.75)	0.87	0.69 (-5.91, 7.28)	0.84
Motor score	102.9 (13.5)	98.5 (19.0)	4.24 (-1.17, 9.64)	0.12	4.80 (-0.58, 10.19)	0.08
Body size at 2-year follow-up						
Weight						
Weight, kg	12.8 (1.9)	12.8 (2.0)	-0.02 (-0.66, 0.61)	0.94	-0.14 (-0.79, 0.51)	0.68
Weight Z score	0.04 (1.3)	0.1 (1.3)	-0.09 (-0.50, 0.33)	0.69	-0.15 (-0.6, 0.28)	0.48
Height						
Height, cm	88.6 (4.9)	87.8 (4.3)	0.82 (-0.78, 2.36)	0.30	0.62 (-0.91, 2.16)	0.42
Height Z score	0.2 (1.6)	0.08 (1.1)	0.13 (-0.24, 0.51)	0.49	0.10 (-0.29, 0.50)	0.60
Head circumference						
Head circumference, cm	49.1 (1.8)	48.9 (2.1)	0.19 (-0.34, 0.86)	0.57	0.22 (-0.43, 0.88)	0.50
Head circumference Z score	-0.6 (1.3)	-0.7 (1.6)	0.12 (-0.37, 0.61)	0.63	0.13 (-0.39, 0.65)	0.62
Use of health services since discharge						
Any hospital admission	38/82 (46.3%)	36/76 (47.4%)	0.95 (0.51, 1.79)	0.90	0.91 (0.44, 1.91)	0.81
Re-admission for respiratory illness	32/82 (39.0%)	28/76 (36.8%)	1.09 (0.58, 2.09)	0.78	1.30 (0.60, 2.81)	0.50
Physiotherapy	21/82 (25.6%)	16/76 (21.1%)	1.29 (0.61, 2.71)	0.50	1.57 (0.69, 3.57)	0.28
Occupational therapy	13/82 (15.9%)	8/76 (10.5%)	1.60 (0.62, 4.11)	0.33	1.65 (0.59, 4.58)	0.34
Speech pathology	14/82 (17.1%)	16/76 (21.1%)	0.77 (0.35, 1.71)	0.52	0.84 (0.37, 1.94)	0.69
Special education	2/82 (2.4%)	0/76 (0.0%)	-	1.00 *	-	-
Psychology	1/82 (1.2%)	1/76 (1.3%)	-	1.00 *	-	-
Organised play group††	8/82 (9.8%)	11/76 (14.5%)	0.64 (0.24, 1.68)	0.37	0.62 (0.23, 1.70)	0.36
Asthma or wheezing	16/82 (19.5%)	20/76 (26.3%)	0.68 (0.32, 1.43)	0.31	0.63 (0.27, 1.49)	0.29
Blood pressure						
Systolic blood pressure						
Mean blood pressure, mmHg	96.6 (10.7)	96.7 (10.5)	-0.07 (-4.48, 4.34)	0.97	-0.46 (-5.41, 4.48)	0.85
Z score	0.7 (1.0)	0.8 (1.0)	-0.06 (-0.49, 0.37)	0.79	-0.08 (-0.56, 0.39)	0.73
Systolic hypertension	9/50 (18.0%)	5/42 (11.9%)	1.62 (0.50, 5.29)	0.42	1.70 (0.46, 6.29)	0.42
Diastolic blood pressure						
Mean blood pressure, mmHg	60.1 (12.5)	60.1 (7.4)	0.03 (-4.32, 4.38)	0.99	-0.26 (-4.96, 4.44)	0.91
Z score	1.4 (1.1)	1.4 (0.7)	-0.003 (-0.39, 0.39)	0.99	-0.05 (-0.47, 0.37)	0.83
Diastolic hypertension	19/50 (38.0%)	17/42 (40.5%)	0.90 (0.39, 2.09)	0.81	0.81 (0.29, 2.31)	0.70
Hypertension	22/50 (44.0%)	18/42 (42.9%)	1.05 (0.46, 2.40)	0.91	1.01 (0.36, 2.85)	0.98
Children behaviour checklist						
Total score	32.3 (23.5)	32.5 (23.1)	0.22 (-7.34, 7.79)	0.95	-2.34 (-9.83, 5.16)	0.54
Total score within clinical range	23/76 (30.3%)	19/72 (26.4%)	1.21 (0.59, 2.48)	0.60	0.91 (0.40, 2.09)	0.83

Drug group data are n (%) or mean (SD). Treatment effects are odds ratios (95% CI) or mean differences (95% CI) unless otherwise indicated. Adjustments were made for study hospital, gestational age at study entry, infant sex, language spoken at home, and mother’s highest education level, unless otherwise specified. BSID-III = Bayley scales of infant development-III.

*Fisher’s exact test.

§ Severe cerebral palsy was defined as unlikely ever to walk (equivalent to GMFCS levels 4 and 5), moderate cerebral palsy was defined as not walking at 2 years, but likely to become ambulant (equivalent to GMFCS levels 2 or 3); and mild cerebral palsy was defined as walking at 2 years (equivalent to GMFCS level 1).

¶Severe developmental delay was defined as a standardised BSID-III score of more than 3 SD below the mean; moderate developmental delay was defined as a standardised BSID-III score of more than 2 SD to 3 SD below the mean; and mild developmental delay was defined as a standardised BSID-III score of more than 1 SD to 2 SD below the mean. Treatment effects are odds ratios (95% CI) from separate logistic models (i.e., any developmental delay vs. none; moderate or severe developmental delay vs. none or mild; and severe developmental delay vs. none, mild, or moderate) or Fisher’s exact test if the outcome is rare.

||Motor developmental delay was adjusted for study hospital, gestational age at entry, number of fetuses, language spoken at home, and mother’s highest education level only.

††Organised playgroup data were adjusted for gestational age at entry, number of fetuses, the infant’s sex, language spoken at home, and mother’s highest education level only.

Infant secondary outcomes before hospital discharge were similar between treatment groups (Table 3). There was a similar incidence of RDS (aOR 1.32, 95% CI 0.64 to 2.76, p = 0.45). Gestational age and body size at birth (weight, length, and head circumference) did not differ between the two treatment groups. Overall, 10.1% of infants were born extremely pre-term, 25% very pre-term, 46.4% moderate to late pre-term and 18.5% at term.

**Table 3:** Secondary outcomes for live born infants assessed before hospital discharge.

	Dexamethasone N = 86	Betamethasone N = 82	Unadjusted treatment effect (95% CI)	Unadjusted P-value	Adjusted treatment effect (95% CI)	Adjusted P-value
Gestational age at birth, weeks	33.2 (3.6)	33.2 (3.9)	-0.003 (-1.14, 1.13)	1.00	-0.08 (-1.18, 1.03)	0.89
Extremely pre-term (<28 weeks)	7 (8.1%)	10 (12.2%)	0.64 (0.23, 1.76)	0.39	0.66 (0.22, 2.00)	0.46
Very pre-term (28 to <32 weeks)	24 (27.9%)	18 (22.0%)	1.38 (0.68, 2.78)	0.37	1.56 (0.74, 3.30)	0.24
Moderate–late pre-term (32 to <37 weeks)	41 (47.7%)	37 (45.1%)	1.11 (0.60, 2.03)	0.74	1.03 (0.53, 2.01)	0.93
Term (≥37 weeks)	14 (16.3%)	17 (20.7%)	0.74 (0.34, 1.63)	0.46	0.70 (0.31, 1.60)	0.40
Any intraventricular haemorrhage	9 (10.5%)	4 (4.9%)	-	0.25 *	-	-
Severe intraventricular haemorrhage (grade 3 or 4)	1 (1.2%)	1 (1.2%)	-	1.00 *	-	-
Periventricular leukomalacia	0 (0.0%)	0 (0.0%)	-	-	-	-
Retinopathy of prematurity requiring treatment	0 (0.0%)	2 (2.4%)	-	0.24 *	-	-
Patent ductus arteriosus	8 (9.3%)	4 (4.9%)	-	0.37*	-	-
Necrotising enterocolitis	2 (2.3%)	3 (3.7%)	-	0.68*	-	-
Neonatal respiratory distress syndrome	31 (36.1%)	27 (32.9%)	1.15 (0.61, 2.17)	0.67	1.32 (0.64, 2.76)	0.45
Severity of respiratory disease†						
Mild	14 (16.3%)	15 (18.3%)	1.15 (0.61, 2.17)	0.67	1.32 (0.64, 2.76)	0.45
Moderate	7 (8.1%)	8 (9.8%)	1.44 (0.64, 3.23)	0.38	1.73 (0.68, 4.40)	0.25
Severe	10 (11.6%)	4 (4.9%)	-	0.16*	-	-
Chronic lung disease	11 (12.8%)	8 (9.8%)	1.36 (0.54, 3.56)	0.54	1.36 (0.48, 3.83)	0.56
Mechanical ventilation	23 (26.7%)	18 (22.0%)	1.30 (0.64, 2.64)	0.47	1.49 (0.67, 3.32)	0.33
Proven infection in first 48 h	0 (0.0%)	0 (0.0%)	-	-	-	-
Infection after first 48 h	5 (5.8%)	4 (4.9%)	-	1.00*	-	-
Admitted to neonatal intensive care unit	45 (52.3%)	36 (43.9%)	1.40 (0.76, 2.58)	0.28	1.63 (0.84, 3.22)	0.15
Length of stay in neonatal intensive care unit, days**	9.7 (19.5)	10.0 (21.6)	0.97 (0.46, 2.06)	0.94	0.81 (0.39, 1.70)	0.59
Body size at birth						
Weight, g	1996.5 (776.2)	2005.7 (844.6)	-9.16 (-256.08, 237.76)	0.94	-29.96 (-271.28, 211.37)	0.80
Length, cm	42.7 (4.9)	42.7 (5.6)	0.01 (-1.67, 1.69)	0.99	-0.22 (-1.84, 1.41)	0.79
Head circumference, cm	30.1 (3.4)	29.9 (3.7)	0.24 (-0.90, 1.38)	0.68	0.09 (-1.03, 1.20)	0.88
Z scores at birth						
Weight	-0.07 (1.1)	-0.2 (1.1)	0.16 (-0.17, 0.49)	0.35	0.13 (-0.20, 0.46)	0.44
Length	-0.3 (1.1)	-0.5 (1.0)	0.23 (-0.17, 0.57)	0.19	0.18 (-0.16, 0.53)	0.28
Head circumference	-0.1 (1.2)	-0.4 (1.1)	0.26 (-0.11, 0.62)	0.17	0.22 (-0.15, 0.60)	0.23
Body size at hospital discharge						
Weight	2796.6 (1240.2)	2830.0 (1266.5)	-33.41 (-419.97, 353.16)	0.86	-36.55 (-433.03, 359.93)	0.86
Length	46.2 (3.2)	46.7 (3.1)	-0.51 (-1.56, 0.55)	0.34	-0.56 (-1.54, 0.41)	0.26
Head circumference	33.2 (2.8)	33.0 (1.7)	0.12 (-0.62, 0.86)	0.75	0.15 (-0.61, 0.90)	0.70
Z scores at hospital discharge						
Weight	-0.9 (0.9)	-0.9 (1.1)	0.07 (-0.24, 0.38)	0.65	0.05 (-0.27, 0.38)	0.74
Length	-1.0 (1.3)	-0.9 (1.3)	-0.07 (-0.51, 0.37)	0.76	-0.08 (-0.52, 0.35)	0.70
Head circumference	-0.02 (1.9)	-0.4 (1.3)	0.36 (-0.19, 0.90)	0.20	0.37 (-0.20, 0.93)	0.20

Drug group data are n (%) or mean (SD). Treatment effects are odds ratios (95% CI) or mean differences (95% CI). Analyses were adjusted for study hospital and gestational age at study entry.

*Fisher’s exact test. Z scores were estimated with the UK–WHO growth reference.

†Treatment effects are odds ratios (95% CI) from separate logistic models (i.e., any respiratory disease vs. none; moderate or severe respiratory disease vs. none or mild; and severe respiratory disease vs. none, mild, or moderate).

**: Data presented as mean (SD), and treatment effects are ratios of means from negative binomial regression model.

Secondary child outcomes measured at two years’ corrected age were similar between groups (Table 2). Of note, systolic blood pressure Z scores and number of children within the hypertensive range were similar in infants of women who received a repeat dose(s) of dexamethasone and infants of women who received a repeat dose(s) of betamethasone (adjusted mean difference (aMD) -0.08 mmHg, 95% CI -0.56 to 0.39, p = 0.73 and aOR 1.01, 95% CI 0.36 to 2.85, p = 0.98 respectively).

For the women we found little to no difference in the incidence of infectious morbidities between groups (aOR 1.68, 95% CI 0.79 to 3.61, p = 0.18) (Table 4). There was no difference between women allocated dexamethasone compared to betamethasone in need for induction of labour (aOR 1.09, 95% CI 0.48 to 2.46, p = 0.83), postpartum haemorrhage (aOR 1.68, 95% CI 0.81 to 3.49, p = 0.16), and caesarean section (dexamethasone group 54 of 82 women, 65.9%, betamethasone group 50 of 86 women, 58.1%, aOR 0.79, 95% CI 0.42 to 1.50, p = 0.47).

**Table 4:** Secondary maternal outcomes assessed before hospital discharge, after birth.

	Dexamethasone N = 86	Betamethasone N = 82	Unadjusted treatment effect (95% CI)	Unadjusted P value	Adjusted treatment effect (95% CI)	Adjusted P value
Maternal-infection related morbidities	24 (27.9%)	16 (19.5%)	1.60 (0.78, 3.28)	0.20	1.68 (0.79, 3.61)	0.18
Chorioamnionitis requiring intrapartum antibiotics	3 (3.5%)	5 (6.1%)	-	0.49*	-	-
Use of postnatal antibiotics	23 (26.7%)	15 (18.3%)	1.63 (0.78, 3.40)	0.19	1.71 (0.79, 3.71)	0.17
Induction of labour	16 (18.6%)	14 (17.1%)	1.11 (0.50, 2.45)	0.80	1.09 (0.48, 2.46)	0.83
Caesarean section	50 (58.1%)	54 (65.9%)	0.72 (0.38, 1.34)	0.30	0.79 (0.42, 1.50)	0.47
Postpartum haemorrhage	28 (32.6%)	19 (23.2%)	1.60 (0.81, 3.17)	0.18	1.68 (0.81, 3.49)	0.16
Blood transfusion required	11 (12.8%)	3 (3.7%)		0.05*		
Postnatal length of stay, days^	5.15 (1.9)	5.10 (2.1)	1.01 (0.88, 1.15)	0.91	1.02 (0.89, 1.17)	0.74

Drug group data are n (%). Treatment effects are odds ratios (95% CI) or mean differences (95% CI). Analyses are adjusted for study hospital and gestational age at entry.

*: Fisher’s exact test.

^: Data presented as mean (SD) and the treatment effects are ratio of means derived from log-Poisson regression model.

## Discussion

In this secondary analysis of the ASTEROID Trial assessing the effect of either dexamethasone or betamethasone as repeat ACS in women who remain at risk of preterm birth after an initial course of ACS on maternal, infant, and childhood health outcomes, we found the risk of our primary outcome, death or neurosensory disability in children at age two years (corrected for prematurity) to be similar. We observed no significant difference in infant health outcomes such as respiratory disease and IVH or in later childhood health outcomes. Maternal health outcomes, such as mode of birth and infectious morbidity, were not significantly different between the treatment groups. Given the difference in the incidence rate of several clinical outcomes, further sufficiently powered studies to examine these outcomes are warranted.

Our study is the first analysis, to our knowledge, to assess for differences in infant, childhood, and maternal outcomes between women who received a repeat dose(s) of dexamethasone and women who received repeat dose(s) of betamethasone. Previous randomised trials have evaluated the efficacy of repeat corticosteroids, but only using betamethasone [6, 7]. The ASTEROID Trial compared the overall effects of dexamethasone and betamethasone but did not report the specific effects of repeat dose(s) [8]. Given the use of either dexamethasone or betamethasone is recommended by most clinical practice guidelines [2, 3, 5] and the use of repeat corticosteroids is also advised [2–5], our study results provide further evidence to support these recommendations.

Previous studies have reached conflicting conclusions regarding the use of dexamethasone for women at risk of preterm birth. For example, previous Cochrane reviews of randomised trials and some cohort studies have reported that the use of dexamethasone resulted in a greater risk of chorioamnionitis for women [1], a decreased risk of IVH [9], a greater risk of PVL in infancy [10], and a greater risk of neurosensory impairment in childhood [11]. The ASTEROID Trial observed no difference in these health outcomes when either dexamethasone or betamethasone was used, as did our secondary analysis of the subgroup of participants who received a repeat dose(s) [8]. There was no evidence that clinical outcomes, including but not limited to severe respiratory disease, IVH and motor developmental delay were significantly different between the two study medications. Further studies primarily designed to address these outcomes with sufficient sample size are warranted. Our study results help support the conclusion that the use of dexamethasone or betamethasone does not result in a difference in health outcomes for women and their infants when a repeat dose(s) is indicated.

Of note, the ASTEROID Trial reported a decrease in the rate of caesarean births in women who received dexamethasone compared to women who received betamethasone [8]. Our analysis of those who received a repeat dose(s) showed no difference in rate of caesarean birth between the treatment groups, suggesting that the type of corticosteroid used as a repeat dose(s) does not influence the mode of birth. However, due to our smaller sample size, we may not have had enough power to detect small differences in caesarean births.

Furthermore, the ASTEROID Trial reported a lower systolic blood pressure z scores and the proportion of children within the hypertensive range at two years (corrected for prematurity) amongst children exposed to dexamethasone compared to children exposed to betamethasone [8]. However, in our study, this difference in blood pressure between corticosteroid treatment groups was not seen.

Importantly, our results provide further evidence for clinicians in health care settings that only have access to dexamethasone. Our findings suggest that women and their children will experience a similar likelihood of outcomes such as maternal infectious morbidity and survival free of neurosensory disability at two years of age. Given that dexamethasone is of a lower cost and more widely available, our results are highly applicable to health care systems in low-income and middle-income countries [12].

Our study has several strengths, including that to our knowledge, this is the first study to compare infant, childhood, and maternal outcomes between those who receive a repeat dose(s) of dexamethasone compared to those who receive repeat dose(s) of betamethasone. A further strength includes assessing important perinatal and neurodisability health outcomes that have previously been sparsely reported [1, 9]. Despite our study occurring in hospitals in two high-income countries, our research has broad applicability to countries where the confirmation of dexamethasone’s safety and efficacy is of great importance.

Our study was limited by its small sample size due to our analysis being restricted by the number of women within the ASTEROID Trial who received a repeat dose(s) of ACS. A few families were lost to follow-up and therefore could not be assessed at 2 years corrected age for the primary and other health outcomes. This may have limited our ability to detect differences in rare outcomes. Therefore, the data on rarer infant, childhood, and maternal outcomes should be interpreted with some caution.

## Conclusion

In conclusion, compared with children born to women who received a repeat dose(s) of dexamethasone, there was little to no difference in the likelihood of survival free of neurosensory disability at age two years in children born to women who received a repeat dose(s) of betamethasone. Additionally, no differences were observed in infant, childhood, and maternal health outcomes between treatment groups. These results suggest that dexamethasone and betamethasone may have similar efficacy when used as a repeat dose(s), and can be used to support clinical practice guideline recommendations. Further research is required to confirm these findings.
